# Dual-Engineering Tailored Co_3_O_4_ Hollow Microspheres Assembled by Nanosheets for Boosting Oxygen Evolution Reaction

**DOI:** 10.3390/molecules30102181

**Published:** 2025-05-16

**Authors:** Yinghan Cui, Shiduo Yang, Jianqiang Zhu, Zaidong Wang, Sen Chen, Jian Qi, Huan Wang

**Affiliations:** 1 Hebei Key Laboratory of Flexible Functionals Materials, School of Materials Science and Engineering, Hebei University of Science and Technology, Shijiazhuang 050000, China; cuiyh066@outlook.com (Y.C.); 19832218054@163.com (J.Z.); wanghbkjstu@163.com (Z.W.); neu_cs@163.com (S.C.); 2 State Key Laboratory of Biochemical Engineering, Institute of Process Engineering, Chinese Academy of Sciences, Beijing 100190, China; 3 School of Chemical Engineering, University of Chinese Academy of Sciences, Beijing 100049, China

**Keywords:** oxygen evolution reaction, hollow microspheres, dual-engineering strategy, oxygen vacancies, active crystal facet

## Abstract

The development of efficient, low-cost electrocatalysts for the oxygen evolution reaction (OER) is crucial for advancing sustainable hydrogen production through water splitting. This study presents a dual-engineering strategy to enhance the OER performance of Co_3_O_4_ by synthesizing hollow microspheres assembled from nanosheets (HMNs) with abundant oxygen vacancies and highly active crystal facet exposure. Through a modified one-step hydrothermal process, Co_3_O_4_ HMNs with exposed (111) and (100) crystal facets were successfully fabricated, demonstrating superior OER activity compared to Co_3_O_4_ nanocubes (NCs) with only (100) facet exposure. The optimized Co_3_O_4_-5% HMNs exhibited a low overpotential of 330 mV at 10 mA cm^−2^ and a Tafel slope of 69 mV dec^−1^. The enhanced performance was attributed to the synergistic effects of crystal facet engineering and defect engineering, which optimized the Co-O bond energy, increased the number of active sites, and improved conductivity. The unique hollow structure further facilitated mass transport and prevented nanosheet stacking, exposing more edge sites for catalytic reactions. This work highlights the potential of geometric and electronic structure modulation in designing high-performance OER catalysts for sustainable energy applications.

## 1. Introduction

Amid growing energy crises and environmental pollution, hydrogen energy has regained global attention [1,2,3,4,5]. Electrolytic water hydrogen production technology can not only convert fluctuating wind and solar energy into hydrogen energy but also achieve zero CO_2_ emissions during the hydrogen production process, while the produced hydrogen has a purity of over 99.9% [6,7,8,9,10]. However, the efficiency of hydrogen production by electrolytic water is hindered by the sluggish oxygen evolution reaction (OER) [11,12,13,14,15,16], a complex four-electron-proton process [17,18]. Although commercial catalysts like RuO_2_ and IrO_2_ are effective, their scarcity and high-cost limit large-scale use [19,20,21]. Developing high-performance, low-cost non-precious metal OER catalysts is thus crucial for reducing hydrogen production costs and improving efficiency. Co_3_O_4_, with its tunable electronic structure that is capable of breaking OER scaling relationships, is a promising candidate [22,23,24]. However, challenges such as low intrinsic activity and limited active sites persist [25,26,27]. Addressing these issues requires strategic geometric and electronic structure modulation to enhance catalytic performance. This research aims not only to address the existing problems of Co_3_O_4_-based catalysts but also to assist in developing highly efficient and affordable OER catalysts. These catalysts are expected to accelerate the widespread use of sustainable hydrogen energy.

The fabrication of superstructure platforms, and in particular, hollow structures, can further expose a greater number of catalytic sites, expedite reactant transportation, and enhance the OER performance [28,29]. Moreover, crystal facet engineering and defect engineering modulation can further unlock the potential of materials and enhance their catalytic performance [30,31]. On the one hand, crystal facet engineering focuses on controlling the exposure of specific crystal facets. Different crystal facets possess distinct atomic arrangements and surface energies, resulting in varied chemical reactivities and physical properties [32,33]. By preferentially exposing the most active crystal facets, the catalytic performance of materials can be significantly enhanced. On the other hand, defect engineering involves intentionally introducing defects (such as vacancies, heteroatoms, etc.) into the lattice, which can effectively regulate the electronic structure and increase the activity of active sites [34,35,36].

This study presents a groundbreaking approach to enhance the performance of Co_3_O_4_-based catalysts for the OER. Through the innovative integration of defect engineering and crystal facet engineering into a modified one-step hydrothermal process, Co_3_O_4_ hollow microspheres assembled by nanosheets (HMNs) were successfully synthesized. This unique synthesis method enables precise control over oxygen vacancies and the exposure of highly active crystal facets by simply adjusting the amount and concentration of hydrogen peroxide. The resultant Co_3_O_4_ HMNs, with exposed (111) and (100) crystal facets, demonstrated a remarkable boost in OER activity, outperforming traditional Co_3_O_4_ nanocubes (NCs) with only (100) crystal facet exposure. The superior performance can be attributed to the dual-engineering modulation strategy. On one hand, this strategy optimizes the Co-O bond energy, enhances reactant adsorption, and improves material conductivity, thereby significantly enhancing the intrinsic catalytic activity. On the other hand, it effectively increases the number of active sites on Co_3_O_4_, further enhancing the overall catalytic performance. This novel synthesis of Co_3_O_4_ HMNs not only paves the way for more efficient electrocatalytic water splitting in hydrogen production but also holds great potential for applications in diverse energy-related fields, including metal-air batteries and rechargeable fuel cells.

## 2. Results and Discussion

**Scheme 1:**
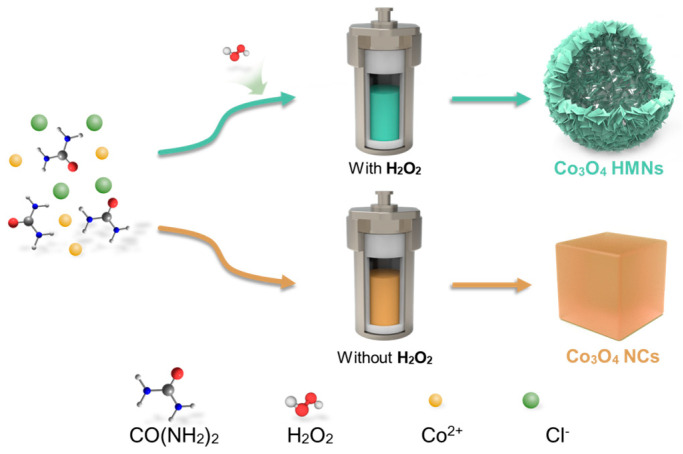
Schematic diagram of the synthesis of Co_3_O_4_ HMNs and Co_3_O_4_ NCs.

Co_3_O_4_ HMNs and Co_3_O_4_ NCs were fabricated via a modified one-step hydrothermal process; the typical synthesis process is displayed in Scheme 1. The key distinction between the synthesis processes of Co_3_O_4_ HMNs and Co_3_O_4_ NCs lies in the strategic introduction of hydrogen peroxide (H_2_O_2_) as a critical reaction modulator (Scheme 1). Specifically, the formation of HMNs relies on the H_2_O_2_-assisted Ostwald ripening mechanism. In the modified one-step hydrothermal process, H_2_O_2_ interacts with cobalt-salt and urea precursors. Its strong oxidizing property oxidizes cobalt ions and controllably dissolves parts of the initial cobalt-containing precursors for oxidative etching. As the reaction proceeds, the dissolved substances accumulate and recrystallize under the reaction temperature conditions. Oxidative-etching-induced local differences make recrystallization likely to occur in the form of hollow structures, with atoms/molecules arranging in an orderly manner to yield nanosheet-assembled hierarchical hollow microspheres on an existing structure. The synergy of oxidative etching and recrystallization provides substances/templates for recrystallization and yields a stable hollow structure, which can prevent nanosheet stacking, expose active sites, offer transport channels, and boost the material’s performance in OER applications [37,38]. In contrast, the NC synthesis pathway deliberately excludes H_2_O_2_, favoring instead a direct nucleation–growth mechanism that produces solid crystalline nanoparticles through conventional thermal decomposition routes.

**Figure 1:**
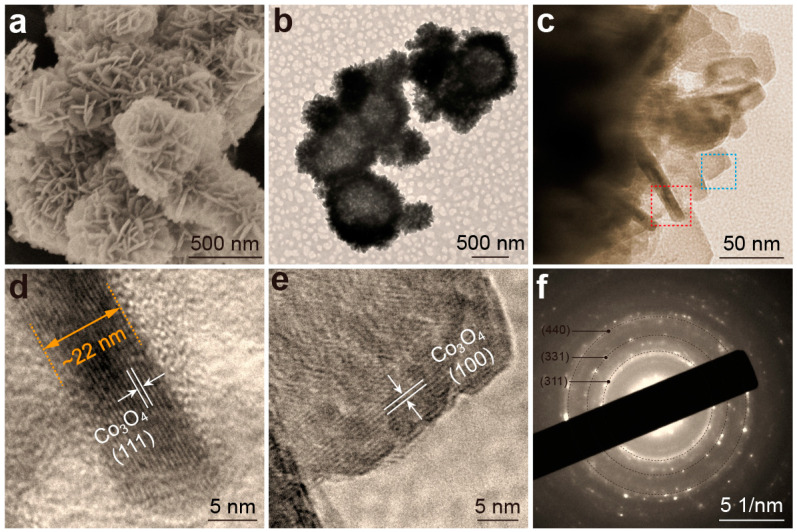
Co_3_O_4_-5% HMNs: (**a**) SEM image; (**b**) TEM image; (**c**) High magnification TEM images; (**d**,**e**) are the HRTEM images corresponding to the parts in the red and blue dashed boxes of Figure 1c, respectively; (**f**) SAED image.

The surface morphology and microstructural characteristics of the synthesized samples were initially investigated using scanning electron microscopy (SEM). Figure 1a and Figure S2a,b present SEM images of Co_3_O_4_ HMNs synthesized from precursor solutions containing different hydrogen peroxide concentrations (3%, 5% and 7%). The SEM images (Figure 1a and Figure S2a,b) reveal that all synthesized samples exhibited well-defined microsphere architectures, which were hierarchically constructed from interconnected nanosheets. Furthermore, transmission electron microscopy (TEM) was employed to observe the internal geometric structure of the synthesized sample. The TEM image (Figure 1b) reveals that the microspheres, assembled from nanosheets, exhibited a hollow structure interspersed with numerous channels. These channels can facilitate efficient mass transport of reactants and products [39,40]. The construction of HMNs can avoid the stacking of Co_3_O_4_ nanosheets, thereby exposing more edge sites and increasing effective contact with the electrolyte [41,42]. Previous research has indicated that edge sites exhibit higher electrocatalytic activity [43]. Furthermore, the construction of HMSs can shorten the electron transfer path, thereby enhancing the OER kinetics [44,45]. Next, an X-ray photoelectron spectroscopy (XPS) survey scan was performed to determine the elemental composition of the sample. The XPS survey spectra (Figure S3) of the Co_3_O_4_-5% HMNs and Co_3_O_4_ NC samples clearly showed the presence of Co and O, as indicated by their characteristic photoelectron peaks. To further observe and measure the shape and size of the nanosheets assembled into microspheres, a TEM image of Co_3_O_4_-5% HMNs was further magnified (Figure 1c). The TEM images (Figure 1c) shows that the nanosheet had a polygonal shape with a thickness of approximately 22 nm. To elucidate the exposed crystal facets of these nanosheets, high-resolution transmission electron microscopy (HRTEM) images were acquired (Figure 1d,e). The HRTEM images (Figure 1d,e) revealed that the lateral facet of the nanosheet was indexed to the (111) crystal facet of Co_3_O_4_, while the basal plane corresponded to the (100) crystal facet of the Co_3_O_4_. These findings not only confirmed the single-crystal nature of the Co_3_O_4_ nanosheet but also revealed that the nanosheet predominantly exposed the (111) and (100) crystal facets, highlighting its well-defined crystallographic orientation. Theoretical and experimental studies have revealed that high-index facets possess a greater density of dangling bonds and higher surface energy, which will contribute to the improvement of catalytic performance [46,47]. In addition, it can be seen from the selected area electron diffraction (SAED) image (Figure 1f) that there were numerous bright spots distributed on the diffraction ring, mainly due to the fact that the selected region included multiple single crystal nanosheets.

**Figure 2:**
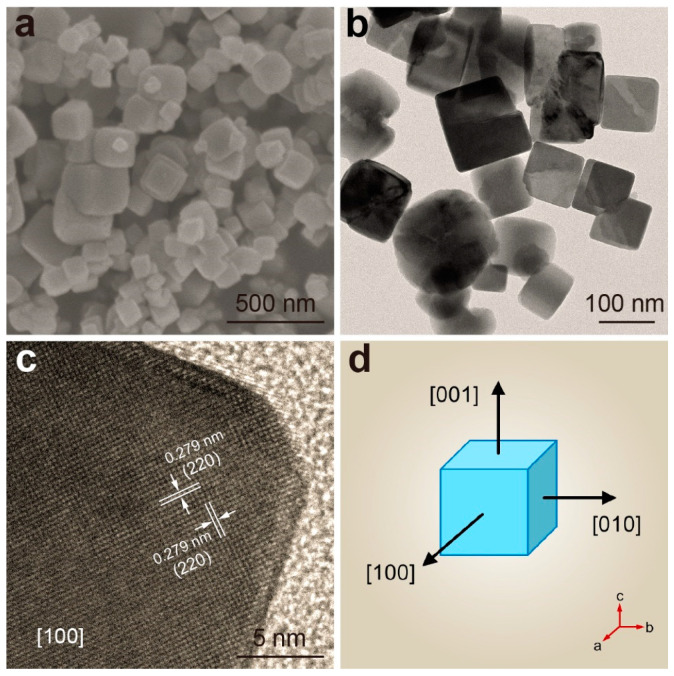
Co_3_O_4_ NCs: (**a**) SEM image; (**b**) TEM images; (**c**) HRTEM image; (**d**) Schematic representation of Co_3_O_4_ NCs; note: [abc] is an index of a specified crystal axis.

As a comparison, Co_3_O_4_ nanocubes (NCs) with only exposed (100) crystal facets were also synthesized. First of all, a precursor solution without added hydrogen peroxide was prepared (Figure 1). Subsequently, Co_3_O_4_ NCs was successfully synthesized through the same hydrothermal process. The SEM image in Figure 2a shows that Co_3_O_4_ NCs had a cubic morphology and a smooth surface. A TEM image (Figure 2b) further revealed that the interior of the cubic nanocrystals was a solid structure with no internal cavities. Due to the symmetry of the structure, Co_3_O_4_ NCs exposed the same (100) crystal facet, which was confirmed by the HRTEM image shown in Figure 2c. Figure 2d presents a schematic diagram of the Co_3_O_4_ NCs derived from the aforementioned analytical characterization data, demonstrating the distinctive architectural features of Co_3_O_4_ NCs. The successful synthesis of Co_3_O_4_ NCs allowed us to undertake a systematic investigation into the facet-dependent properties and their crucial roles in electrocatalytic processes.

**Figure 3:**
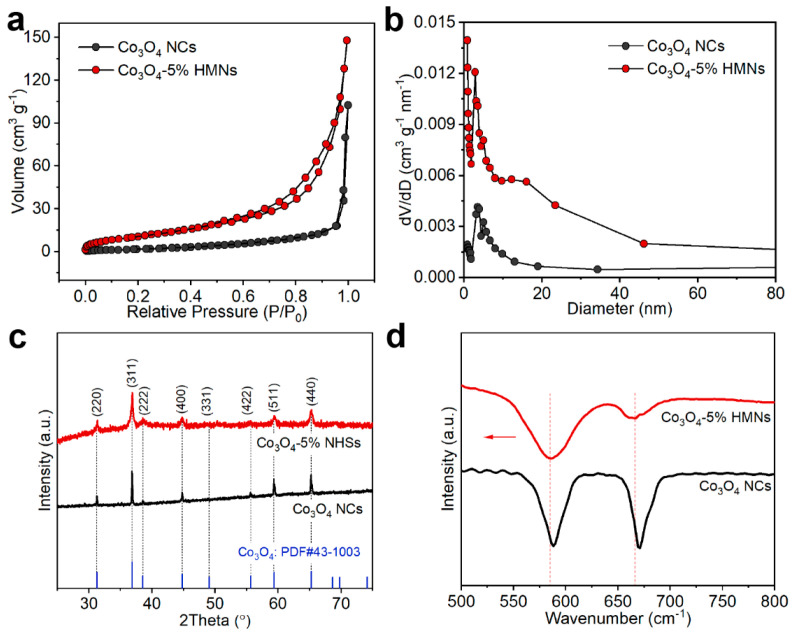
Co_3_O_4_-5% HMNs and Co_3_O_4_ NCs: (**a**) Nitrogen adsorption–desorption isotherms, (**b**) pore distribution curves, (**c**) XRD patterns, (**d**) FTIR spectra.

Due to the fact that HMNs are assembled from a large number of nanosheets, a large number of pores of different sizes form, which play an important role in the diffusion and adsorption of reactants. Therefore, studying the specific surface area and pore distribution of the samples is beneficial for exploring differences in material properties. Next, nitrogen adsorption–desorption measurements were conducted to study the specific surface area and pore distribution of the samples. Figure 3a displayed the nitrogen adsorption–desorption isotherms for both Co_3_O_4_-5% HMNs and Co_3_O_4_ NCs. The specific surface areas were calculated using the Brunauer-Emmett-Teller (BET) method based on the corresponding isotherm data [48]. The results show that Co_3_O_4_ HMNs had a specific surface area of 42.6 m^2^ g^−1^, i.e., larger than Co_3_O_4_ NCs (8.2 m^2^ g^−1^). This indicated that Co_3_O_4_-5% HMNs could expose more effective surfaces to the electrolyte, thereby increasing the probability of contact between active sites and reactants. This result further confirmed the structural advantages of HMNs. Moreover, the pore distribution curves in Figure 3b show that the Co_3_O_4_-5% HMNs had more pores, and these pore sizes were mainly around 5–50 nm, while Co_3_O_4_ NCs had fewer pores, with a pore size range concentrated between 3 and 20 nm. More pores and larger pore sizes facilitate the diffusion of the electrolyte, thereby accelerating the reaction kinetics process. Afterwards, X-ray diffraction (XRD) patterns were collected to further investigate the phase composition and structural characteristics of the cobalt oxides. The XRD patterns presented in Figure 3c and Figure S4 demonstrate that Co_3_O_4_ HMNs and Co_3_O_4_ NCs exhibited diffraction peaks that precisely matched the reference pattern of cubic phase Co_3_O_4_ (PDF#: 43-1003, space group: Fm-3m) [49,50]. This complete correspondence in peak positions and relative intensities confirmed the phase purity of both materials without detectable impurities. Fourier transform infrared spectroscopy (FTIR) measurements were conducted to investigate structural modifications in the materials by analyzing the characteristic vibrational modes of chemical bonds. As shown in Figure 3d, the absorption peaks at ~585 and ~665 cm^−1^ in the FTIR spectra were attributed to the stretching vibration of Co-O bands [51,52]. It could be clearly distinguished from the FTIR spectra that the absorption peak of Co_3_O_4_-5% HMNs had a red shift compared to Co_3_O_4_ NCs. The observed red shift in the absorption peaks of Co_3_O_4_-5% HMNs suggested a significant weakening of Co-O bonds within the spinel structure. This bond weakening induced an upward shift of the O 2p band toward the Fermi level, thereby facilitating enhanced oxygen ion mobility and more efficient oxygen exchange processes [53,54,55]. Consequently, these structural modifications contributed to improving the OER kinetics through accelerated surface redox processes.

**Figure 4:**
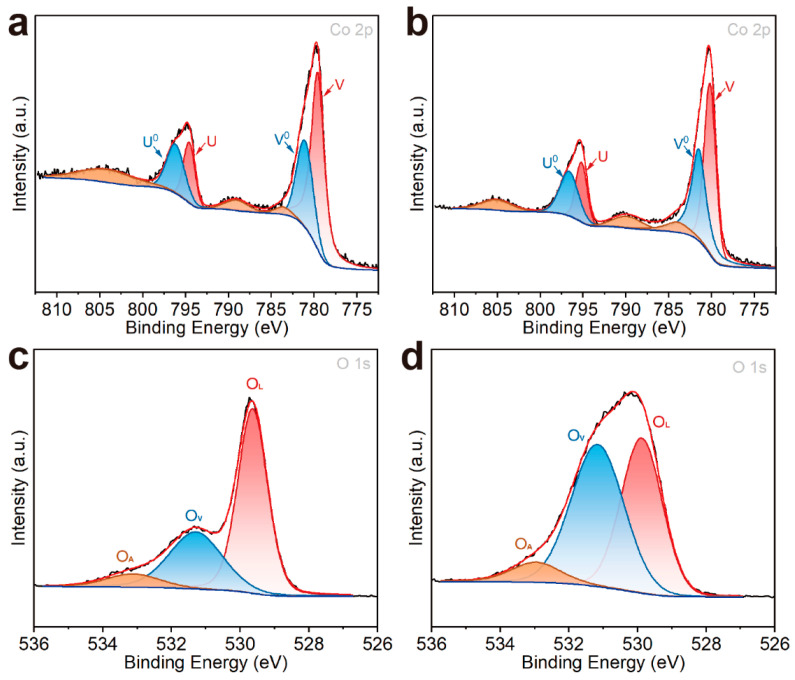
Co 2p XPS spectra of (**a**) Co_3_O_4_ NCs and (**b**) Co_3_O_4_-5% HMNs; O 1s XPS spectra of (**c**) Co_3_O_4_ NCs and (**d**) Co_3_O_4_-5% HMNs.

The electrocatalytic reaction mainly occurs on the surface of a catalyst, so studying the surface properties of a catalyst is of great significance for clarifying the catalytic reaction mechanism. In our study, XPS, as a surface-sensitive technique with a typical probing depth of 3–10 nm, was employed to characterize the chemical environments and electronic structures of surface elements, providing critical insights into the morphological features and defect chemistry of the electrocatalysts. The Co 2p spectra of Co_3_O_4_ NCs and Co_3_O_4_-5% HMNs were further analyzed by peak fitting (Figure 4a,b). In the Co 2p spectrum (Figure 4a) of Co_3_O_4_ NCs, the characteristic absorption peaks at approximately 796.19 eV (u_0_), 781.10 eV (v_0_), 794.59 eV (u), and 779.59 eV (v) were assigned to Co^2+^ 2p_1/2_, Co^2+^ 2p_3/2_, Co^3+^ 2p_1/2_, and Co^3+^ 2p_3/2_, respectively, confirming the coexistence of Co^3+^ and Co^2+^ species [56,57]. Meanwhile, in the Co 2p spectrum of Co_3_O_4_-5% HMNs (Figure 4b), adsorption peaks corresponding to Co^2+^ 2p_1/2_, Co^2+^ 2p_3/2_, Co^3+^ 2p_1/2_, and Co^3+^ 2p_3/2_ were located at 796.63 eV (u_0_), 781.51 eV (v_0_), 795.20 eV (u), and 780.20 eV (v), respectively. It is noteworthy that, through fitting calculations, the ratio of Co^2+^/(Co^2+^+Co^3+^) in Co_3_O_4_ NCs and Co_3_O_4_-5% HMNs was greater than 0.33, due to the presence of oxygen vacancies increasing the content of Co^2+^ [25,58]. Meanwhile, the results also showed that the ratio of Co^2+^/(Co^2+^+Co^3+^) in Co_3_O_4_-5% HMNs (49.2%) was higher than that in Co_3_O_4_ NCs (39.1%), indicating that more oxygen vacancies were generated in Co_3_O_4_-5% HMNs. To further validate this conclusion, the O 1s spectra of both Co_3_O_4_ NCs and Co_3_O_4_-5% HMNs were collected and subjected to peak fitting analysis (Figure 4c,d). As illustrated in Figure 4c,d, the O 1s spectra exhibited three distinct peaks at approximately 533.1 eV (O_A_), 531.3 eV (O_V_), and 529.6 eV (O_L_), which were assigned to oxygen species in surface-adsorbed hydroxyl groups, oxygen vacancies, and metal-oxygen bonds, respectively [59,60]. The fitting results (Table S1) reveal that the relative area of the oxygen vacancy (O_V_) peak in Co_3_O_4_-5% HMNs constituted 49.9% of the total O 1s spectrum, significantly higher than that in Co_3_O_4_ NCs (32.8%). This finding further corroborates that Co_3_O_4_-5% HMNs possessed a higher concentration of oxygen vacancies compared to Co_3_O_4_ NCs. The increased concentration of oxygen vacancies in Co_3_O_4_-5% HMNs primarily originated from unsaturated coordination induced by the exposure of additional edge sites. These vacancies led to a noticeable weakening of Co-O bonds, as evidenced by FTIR spectral analysis [61,62]. In addition, the generation of vacancies could regulate the adsorption of reactants and reaction intermediates and improve the intrinsic reaction activity [53]. Therefore, defect engineering can efficiently modulate the electronic structure of materials, which will contribute to the improvement of OER performance.

**Figure 5:**
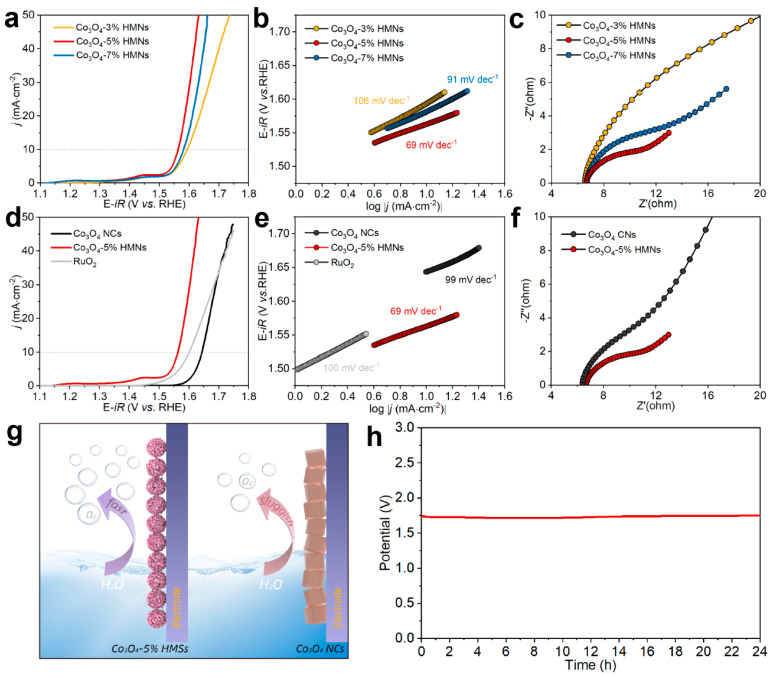
(**a**) polarization curves, (**b**) Tafel plots and (**c**) Nyquist plots of Co_3_O_4_-3% HMNs, Co_3_O_4_-5% HMNs, Co_3_O_4_-7% HMNs; Co_3_O_4_-5% HMNs and Co_3_O_4_-7% HMNs. (**d**) polarization curves and (**e**) Tafel plots of Co_3_O_4_-5% HMNs, Co_3_O_4_ NCs and commercial RuO_2_. (**f**) Nyquist plots of Co3O4-5% HMNs and Co_3_O_4_ NCs. (**g**) Schematic illustration of OER process on Co_3_O_4_-5% HMNs and Co_3_O_4_ NCs. (**h**) chronopotentiometry curve of Co_3_O_4_-5% HMNs for durability test.

The OER performance of the synthesized electrocatalysts was systematically evaluated in a 1.0 M KOH electrolyte using a standard three-electrode system. Initially, linear sweep voltammetry (LSV) was employed to obtain polarization curves, enabling the comparison of overpotentials (η) among different electrocatalysts at specific current densities. A lower overpotential at identical current density signifies superior electrocatalytic performance [63]. Figure 5a presents the polarization curves of Co_3_O_4_ HMNs synthesized from precursor solutions containing varying concentrations of hydrogen peroxide (3%, 5%, and 7%). Notably, Co_3_O_4_-5% HMNs exhibited the lowest overpotential (330 mV) at a current density of 10 mA cm^−2^, while deviations from the optimal hydrogen peroxide concentration (either lower or higher) resulted in increased overpotentials. Furthermore, Tafel plots were constructed to analyze the rate of current density increase with respect to overpotential, where a smaller Tafel slope indicated faster kinetics. As shown in Figure 5b, Co_3_O_4_-5% HMNs demonstrated the smallest Tafel slope compared to Co_3_O_4_-3% HMNs and Co_3_O_4_-7% HMNs, confirming that the addition of 5% hydrogen peroxide to the precursor solution optimally enhanced the OER performance of Co_3_O_4_-5% HMNs. The enhanced OER performance of Co_3_O_4_-5% HMNs was inherently linked to its structure and properties. Next, electrochemical impedance spectroscopy (EIS) testing was conducted to assess the interfacial charge transfer capabilities of the catalysts. In the Nyquist plot, the diameter of the semicircle observed in the high-frequency region corresponds to the charge transfer resistance (R_ct_), which reflects the kinetics of electron transfer at the electrode-electrolyte interface [64]. As shown in Figure 5c, Co_3_O_4_-5% HMNs exhibited a significantly smaller R_ct_ compared to Co_3_O_4_-3% HMNs and Co_3_O_4_-7% HMNs, demonstrating superior charge transfer kinetics and enhanced electrocatalytic efficiency. Afterwards, the surface vacancy defects of these HMNs were investigated through XPS analysis. For this purpose, O 1s spectra of Co_3_O_4_-3% HMNs and Co_3_O_4_-7% HMNs were collected, followed by spectral deconvolution (Figure S5). Table S1 summarizes the ratio of O_v_ derived from the deconvolution analysis of O 1s XPS spectra (Figure 5d and Figure S5). As evident from Table S1, as the concentration of hydrogen peroxide increased, the oxygen vacancy concentration in Co_3_O_4_ HMNs increased. The above results are primarily attributed to the following: while an appropriate concentration of oxygen vacancies effectively enhances the number of active sites and improves electrical conductivity, excessive oxygen vacancies conversely lead to a deterioration in the material’s conductive properties [65,66,67]. To investigate the influence of crystal facets on OER performance, Co_3_O_4_ NCs with exclusively exposed (111) facets were synthesized (Figure 2). In comparison, Co_3_O_4_-5% HMNs exhibited a lower overpotential of 330 mV at 10 mA cm^−2^ than Co_3_O_4_ NCs and commercial RuO_2_, as clearly demonstrated in Figure 5d. Meantime, the Tafel plots in Figure 5e also reveal that Co_3_O_4_-5% HMNs possessed a smaller Tafel slope (69 mV dec^−1^) than Co_3_O_4_ NCs (99 mV dec^−1^) and commercial RuO_2_ (100 mV dec^−1^). EIS testing (Figure 5f) revealed that Co_3_O_4_-5% HMNs exhibited a lower R_ct_, suggesting excellent charge transfer kinetics, which contributed substantially to the enhanced electrochemical performance (Figure 5g). More significantly, previous characterizations have also revealed that Co_3_O_4_-5% HMNs have more oxygen vacancies and weakened Co-O bands compared to Co_3_O_4_ NCs, which undoubtedly contributed to the enhanced OER performance. Mass activity, which accounts for the influence of catalyst mass, is another critical metric for evaluating OER performance. As depicted in Table S2, Co_3_O_4_-5% HMNs achieved a high mass activity of 13.39 A g^−1^ at an overpotential of 350 mV, which was higher than those of Co_3_O_4_-3% HMNs (5.84 A g^−1^), Co_3_O_4_-7% HMNs (13.39 A g^−1^) and Co_3_O_4_ NCs (0.59 A g^−1^). Additionally, the turnover frequency (TOF), representing the number of oxygen molecules produced per active site per unit time, provided insights into the intrinsic activity of individual catalytic sites. It can be concluded from Table S3 that Co_3_O_4_-5% HMNs obtained the highest TOF value (11.28 × 10^−3^ s^−1^) among all synthesized electrocatalysts. Compared with cobalt-based electrocatalysts reported in recent literature, this catalyst demonstrated outstanding OER performance (Table S4). Finally, chronopotentiometry measurements were performed to evaluate the electrochemical stability of the materials [63]. As illustrated in Figure 5h, Co_3_O_4_-5% HMNs maintained stable OER performance for up to 24 h with almost no degradation, demonstrating their excellent durability. To investigate whether the electrocatalyst underwent changes after the reaction, TEM and FTIR were used to characterize its morphology and structure. As clearly demonstrated in Figure S6, the TEM image of post-reaction Co_3_O_4_-5% HMNs revealed remarkable morphological stability, with the catalyst maintaining its pristine structural characteristics without significant alteration. The FTIR spectra (Figure S7) reveals that the characteristic absorption peaks corresponding to the Co-O vibrational mode in the post-reaction Co_3_O_4_-5% HMNs catalyst remained essentially unchanged, suggesting excellent structural integrity and stability of the catalyst framework throughout the electrochemical process.

Based on the above discussion, the outstanding OER performance of Co_3_O_4_-5% HMNs can be primarily attributed to the regulation of dual-engineering strategies and the construction of unique hollow structure. Firstly, the Co_3_O_4_ nanosheet structure could expose high-index (111) crystal facets, which possessed the biggest dangling bond density and highest surface energy. A higher density of dangling bonds implies a greater number of coordination-unsaturated atoms, which, in turn, serves as a more robust indication of the existence of a larger quantity of oxygen vacancies. The increase in oxygen vacancy content could not only weaken Co-O bonds but also regulate the adsorption of reactants and reaction intermediates. The weakening of this bond caused the O 2p band to shift upward toward the Fermi level. Therefore, the mobility of oxygen ions was enhanced, and the oxygen exchange processes became more efficient. Secondly, self-assembling these highly active nanosheets into HMNs can effectively prevent the aggregation and stacking of Co_3_O_4_ nanosheets, thus exposing more edge sites and enhancing the effective contact area with the electrolyte. Thus, the edge sites were more fully exposed, significantly increasing the number of coordination-unsaturated atoms. Furthermore, when nanosheets were assembled into hollow structures, an extensive channel network formed, facilitating electrolyte flow. This significantly expanded the contact area between the structure and the electrolyte, enabling more efficient interaction during electrolysis. All in all, the dual-engineering strategy effectively regulated the electronic structure and micro-geometric structure of Co_3_O_4_-5% HMNs, synergistically enhancing their OER performance.

## 3. Materials and Methods

All chemicals, i.e., cobalt chloride hexahydrate (CoCl_2_·6H_2_O), urea ((NH_2_)_2_CO), hydrogen peroxide (H_2_O_2_, 30 wt%), potassium hydroxide (KOH), anhydrous ethanol (C_2_H_5_OH, ≥99.7%), and deionized water (H_2_O, 18.2 MΩ·cm^−1^ at 25 °C), were of analytical grade and used as received without further purification. The Nafion solution (5 wt%) was obtained from commercial sources.

Co_3_O_4_ HMNs: Firstly, 0.136 g of hexahydrate cobalt chloride and 0.173 g of urea were sequentially dissolved in 64 mL of deionized water to form a homogeneous and transparent purple red solution. Secondly, a certain amount of hydrogen peroxide solution with the mass fraction of hydrogen peroxide (H_2_O_2_) in the total solution being 3%, 5%, and 7%, respectively, was added to the above solution to form the final precursor solution. Next, the prepared precursor solutions were transferred to 100 mL stainless steel reaction vessels. The stainless-steel reaction vessels were placed in a drying oven at 160 °C for 20 h and then naturally cooled to room temperature in air. After the reaction, the suspension was separated by centrifugation to obtain a precipitate. These precipitates were then washed with deionized water and ethanol several times. Finally, the washed sample was dried in an 80 °C vacuum oven for 12 h to obtain a black/brown powder, which was Co_3_O_4_ HMNs. According to the amount of hydrogen peroxide added, the final electrocatalysts obtained were named Co_3_O_4_-3% HMNs, Co_3_O_4_-5% HMNs, and Co_3_O_4_-7% HMNs. 

Co_3_O_4_ nanocubes (NCs): The synthesis of Co_3_O_4_ NCs was similar to that of Co_3_O_4_ HMNs, except that no hydrogen peroxide solution was added during the preparation of the precursor solution.

Scanning electron microscopy (SEM) images were acquired by means of a JSM-6700 (JEOL, Akishima, Tokyo, Japan) scanning electron microscope operating at an accelerating voltage of 5.0 kV. Transmission electron microscopy (TEM) images, high-resolution transmission electron microscopy (HRTEM) images, and selected area electron diffraction (SAED) images were obtained using a JEM-2100F (JEOL, Akishima, Tokyo, Japan) transmission electron microscope, which was operated at an accelerating voltage of 200.0 kV. Nitrogen adsorption–desorption measurements were conducted using a Micromeritics ASAP 2020 analyzer (Micromeritics Instrument Corporation, Norcross, GA, USA) to determine the specific surface area and pore size distribution through Brunauer-Emmett-Teller (BET) and Barrett-Joyner-Halenda (BJH) methods, respectively. The crystalline structure was characterized by X-ray powder diffraction (XRD) using a Bruker D8 Advance diffractometer (Bruker AXS, Karlsruhe, Germany) with Cu Kα radiation (λ = 1.5406 Å) at 40 kV and 40 mA. X-ray photoelectron spectroscopy (XPS) was collected by a Thermo K-Alpha+ X-ray photoelectron spectrometer. Fourier transform infrared spectra (FTIR) were obtained by a Perkin Elmer spectrum 100 infrared spectrometer.

The working electrode was prepared through the following procedure: Initially, 5.0 mg of the electrocatalyst was ultrasonically dispersed in 500 μL ethanol for 10 min to achieve a uniform suspension. Subsequently, 30 μL of 5 wt% Nafion solution was added to the suspension, followed by additional sonication for 5 min to ensure complete mixing and formation of a homogeneous catalyst ink. Finally, 20 μL of the optimized catalyst ink was precisely deposited onto a polished glassy carbon electrode (5 mm in diameter) using a micropipette and allowed to dry at room temperature, resulting in a uniform catalyst layer ready for electrochemical testing (Figure S1). In this work, all electrochemical tests were conducted in 1.0 M KOH solution using a three-electrode configuration at ambient temperature on a CHI660E electrochemical workstation. The three-electrode system comprised a graphite electrode used as the counter electrode, a mercury oxide electrode (Hg/HgO) as the reference electrode, and a glassy carbon electrode coated with the electrocatalyst as the working electrode. Polarization curves were collected by linear sweep voltammetry (LSV) at a scan rate of 10 mV s^−1^, with the potential ranging from 0 to 0.8V versus Hg/HgO. These LSV curves were subsequently transformed into Tafel plots by graphing overpotential (η) against the logarithm of current density (log|j|). Electrochemical impedance spectroscopy (EIS) measurements were conducted over a frequency range of 100 kHz to 1 Hz at a fixed potential of 0.55 V versus Hg/HgO. Additionally, the electrochemical double-layer capacitance (C_dl_) was determined using cyclic voltammetry (CV) at scan rates of 10, 30, 50, 70, and 90 mV s^−1^ within a potential window of −0.1 to 0.0 V versus Hg/HgO, ensuring the absence of faradaic processes.

The turnover frequency (TOF) serves as a critical metric for assessing the intrinsic activity of catalysts, providing deeper insights into their OER performance [2,3]. The TOF value quantifies the number of oxygen molecules generated per active site per unit time and can be calculated using the following equation:

(1) $${\mathrm{TOF}(s}^{-1})=\frac{j\times M_{W,\mathrm{catalyst}}}{F\times n\times m_{\mathrm{catalyst}}}$$

where j represents the current density at a given overpotential of 350 mV (mA cm^−2^), MW, catalyst is the molar mass of the synthesized electrocatalyst (g mol^−1^), F is the Faraday constant (F = 96,485 C mol^−1^), n is the number of electrons transferred during the OER process (n = 4), and m is the mass loading of the electrocatalyst per geometric electrode area (mg cm^−2^).

## 4. Conclusions

In conclusion, this study innovatively synthesized Co_3_O_4_ hollow microspheres assembled from nanosheets (HMNs) via a dual-engineering strategy, precisely tailoring crystal facet exposure and introducing oxygen vacancies. The Co_3_O_4_-5% HMNs achieved remarkable OER performance, requiring only 330 mV overpotential at 10 mA cm^−2^ and featuring a Tafel slope of 69 mV dec^−1^, outperforming both Co_3_O_4_ nanocubes (NCs) and commercial RuO_2_. The outstanding catalytic activity resulted from crystal facet engineering exposing high-index (111) facets with high surface energy and defect engineering optimizing Co-O bond energy and reactant adsorption through oxygen vacancies, while the unique hollow structure of HMNs prevented nanosheet stacking, maximized edge site exposure, and facilitated efficient mass transport to accelerate OER kinetics. This research clarifies the structure/performance relationship of Co_3_O_4_-based electrocatalysts and presents an effective approach for developing high-performance, low-cost OER catalysts, which are vital for sustainable hydrogen production, with a scalable synthesis method. The potential application of this approach in other catalytic systems is worthy of further exploration.

## Data Availability

The original contributions presented in this study are included in the article/Supplementary Materials. Further inquiries can be directed to the corresponding authors.

## Supplementary Materials

The following supporting information can be downloaded at: https://www.mdpi.com/article/10.3390/molecules30102181/s1, Figure S1. The polarization curves for the Co_3_O_4_-5% HMNs with different loading (i. 0.722 mg cm^−2^, ii. 0.963 mg cm^−2^, iii. 1.203 mg cm^−2^).; Figure S2. SEM images of (a) Co_3_O_4_-3% HMNs and (b) Co_3_O_4_-7% HMNs.; Figure S3. XPS full spectra of Co_3_O_4_-5% HMNs and Co_3_O_4_ NCs.; Figure S4. XRD patterns of Co_3_O_4_-3% HMNs, Co_3_O_4_-5% HMNs and Co_3_O_4_-7% HMNs.; Figure S5. O 1s spectra of Co_3_O_4_-3% HMNs and Co_3_O_4_-7% HMNs.; Figure S6. TEM image of Co_3_O_4_-5% HMNs after OER.; Figure S7. FTIR spectra of Co_3_O_4_-5% HMNs before and after OER.; Table S1. The ratio of Ov obtained from O 1s spectra of Co_3_O_4_-3% HMNs, Co_3_O_4_-5% HMNs, Co_3_O_4_-7% HMNs and Co_3_O_4_ NCs.; Table S2. The mass activity of samples.; Table S3. The TOF of samples.; Table S4. Comparison with OER performance of the electrocatalysts in literature [68,69,70,71,72,73,74,75,76,77,78,79].
