# Supplementary material for: Dual-Engineering Tailored Co3O4 Hollow Microspheres Assembled by Nanosheets for Boosting Oxygen Evolution Reaction
Source: Molecules. 2025 May 16;30(10):2181. doi: 10.3390/molecules30102181 (PMC12113657; doi:10.3390/molecules30102181)
Supplement: Supplementary file 1 [file molecules-30-02181-s001.zip › molecules-3629381-supplementary.pdf]

Supplementary Material

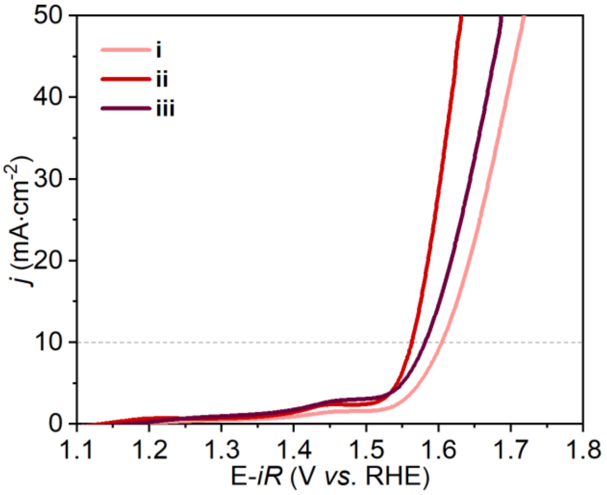

Figure S1. The polarization curves for the Co_3O_4-5% HMNs with different loading (i. 0.722 mg cm^{-2}, ii. 0.963 mg cm^{-2}, iii. 1.203 mg cm^{-2}).

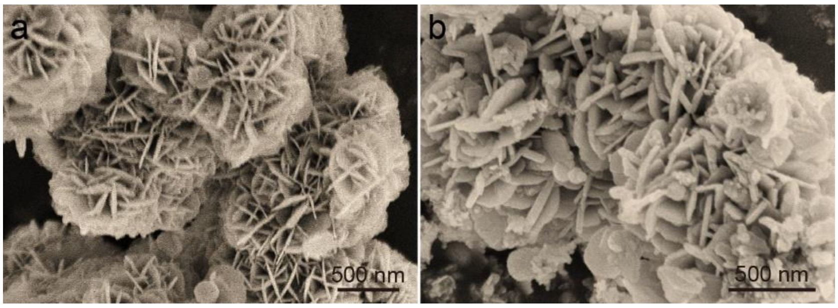

Figure S2. SEM images of (a) Co_3O_4-3% HMNs and (b) Co_3O_4-7% HMNs.

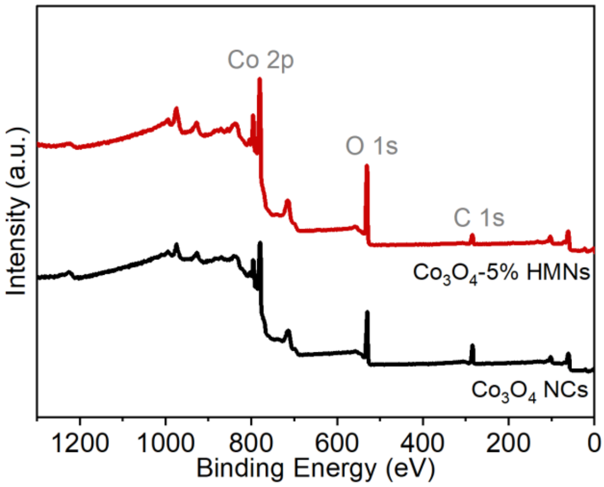

Figure S3. XPS full spectra of Co_3O_4-5% HMNs and Co_3O_4 NCs.

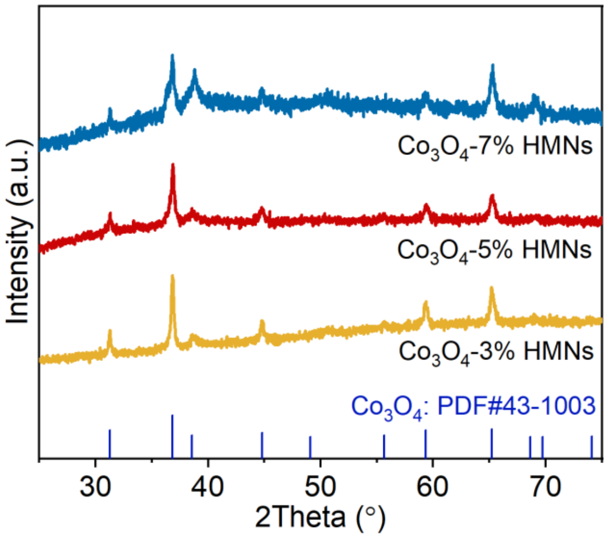

Figure S4. XRD patterns of Co_3O_4-3% HMNs, Co_3O_4-5% HMNs and Co_3O_4-7% HMNs.

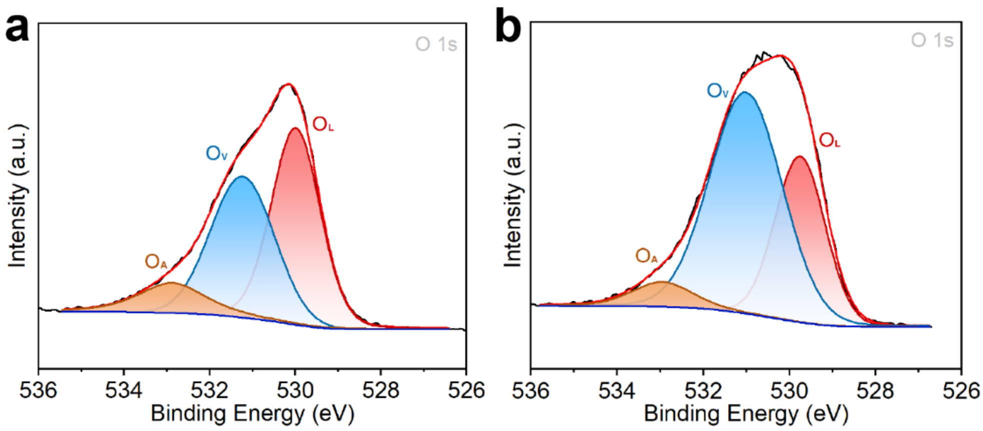

Figure S5. O 1s spectra of Co_3O_4-3% HMNs and Co_3O_4-7% HMNs.

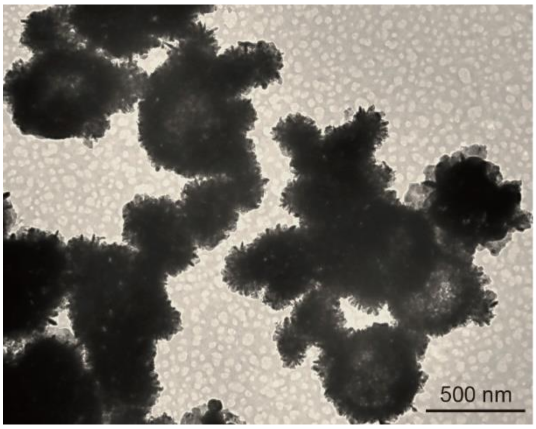

Figure S6. TEM image of Co_3O_4-5% HMNs after OER.

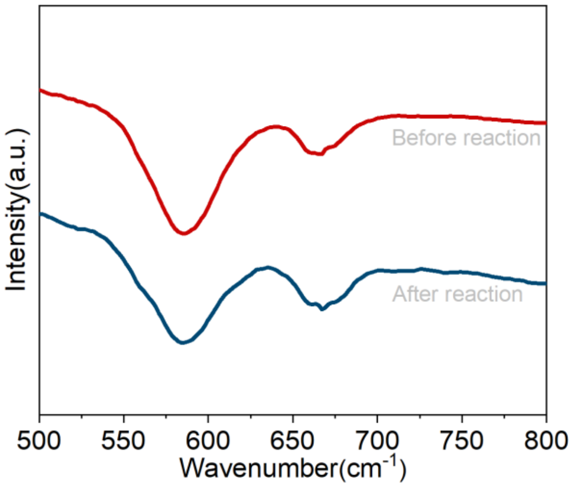

Figure S7. FTIR spectra of Co_3O_4-5% HMNs before and after OER.

Table S1. The ratio of O_v obtained from O 1s spectra of Co_3O_4-3% HMNs, Co_3O_4-5% HMNs, Co_3O_4-7% HMNs and Co_3O_4 NCs.

Sample	O_v ratio/%
Co_3O_4-3% HMNs	41.3
Co_3O_4-5% HMNs	49.9
Co_3O_4-7% HMNs	61.9
Co_3O_4 NCs	32.8

Table S2. The mass activity of samples.

Sample	Mass activity (A g^{-1})
Co_3O_4-3% HMNs	5.84
Co_3O_4-5% HMNs	13.39
Co_3O_4-7% HMNs	7.52
Co_3O_4 NCs	0.59

Table S3. The TOF of samples.

Sample	$TOF_{mass}\times10^{-3}/s^{-1}$
Co_3O_4-3% HMNs	4.92
Co_3O_4-5% HMNs	11.28
Co_3O_4-7% HMNs	6.33
Co_3O_4 NCs	0.49

Table S4. Comparison with OER performance of the electrocatalysts in literature.

Materials	Electrolyte	Overpotential (mV)	Tafel slope (mV·dec^{-1})	Ref.
Co/CoP-5	1.0 M KOH	340	79.5	[S1]
Co_3O_4 microplates	1.0 M KOH	385	56	[S2]
Co_3O_4	1.0 M KOH	421	69.5	[S3]
$Co(OH)_2$ nanosheets	1.0 M KOH	387	94	[S4]
Co_3O_4/CC	1.0 M KOH	440	114	[S5]
P-Co_3O_4 NSs	1.0 M KOH	330	96	[S6]
$NiCo_2O_4$/Ti	1.0 M KOH	353	61	[S7]
Co_3O_4-Cu@B	1.0 M KOH	360	88	[S8]
V(III)-$NiCo_2O_4$	1.0 M KOH	344	72	[S9]
C-Co/Co_3O_4	1.0 M KOH	352	80	[S10]
P-CoS	1.0 M KOH	340	73	[S11]
Co_3O_4/$NiCo_2O_4$	1.0 M KOH	~400	110	[S12]
Co_3O_4-5% HMNs	1.0 M KOH	330	69	This work

References:

[S1] Z.H. Xue, H. Su, Q.Y. Yu, B. Zhang, H.H. Wang, X.H. Li, J.S. Chen, Janus Co/CoP nanoparticles as efficient mott-schottky electrocatalysts for overall water splitting in wide pH range, Adv. Energy Mater. 7 (2017) 1602355.

[S2] H. Liu, F. Ma, C. Xu, L. Yang, Y. Du, P. Wang, S. Yang, L. Zhen, Sulfurizing-induced hollowing of Co_9S_8 microplates with nanosheet units for highly efficient water oxidation, ACS Appl. Mater. Inter. 9 (2017) 11634–11641.

[S3] R. Zhang, W. Ke, S. Chen, X. Yue, Z. Hu, T. Ning, Phase evolution of vulcanized Co_3O_4 catalysts during oxygen evolution reaction, Appl. Surf. Sci. 546 (2021) 148819.

[S4] B. Malik, S. Anantharaj, K. Karthick, D. K. Pattanayak, S. Kundu, Magnetic CoPt nanoparticle-decorated ultrathin $Co(OH)_2$ nanosheets: An efficient bi-functional water splitting catalyst, Catal. Sci. Technol. 7 (2017) 2486–2497.

[S5] G. Yang, H. Xiang, M. Rauf, H. Mi, X. Ren, P. Zhang, Y. Li, Plasma enhanced atomic-layer-deposited nickel oxide on Co_3O_4 arrays as highly active electrocatalyst for oxygen evolution reaction, J. Power Sources 481 (2021) 228925.

[S6] Y. Lu, C. Li, Y. Zhang, X. Cao, G. Xie, M. Wang, D. Peng, K. Huang, B. Zhang, T. Wang, W. Sheng, Y. Huang, Engineering of cation and anion vacancies in Co_3O_4 thin nanosheets by laser irradiation for more advancement of oxygen evolution reaction, Nano Energy 83 (2021) 105800.

[S7] W. Bao, L. Xiao, J. Zhang, P. Jiang, X. Zou, C. Yang, X. Hao, T. Ai, Electronic and structural engineering of $NiCo_2O_4$/Ti electrocatalysts for efficient oxygen evolution reaction, Int. J. Hydrogen Energ. 46 (2021) 10259–10267.

[S8] A. Saad, D. Liu, Y. Wu, Z. Song, Y. Li, T. Najam, K. Zong, P. Tsiakaras, X. Cai, Ag nanoparticles modified crumpled borophene supported Co_3O_4 catalyst showing superior oxygen evolution reaction (OER) performance, Appl. Catal. B-Environ. 298 (2021) 120529.

[S9] X. Wang, Y. Zhou, J. Luo, F. Sun, J. Zhang, Synthesis of V-doped urchin-like NiCo2O4 with rich oxygen vacancies for electrocatalytic oxygen evolution reactions, Electrochim. Acta 406 (2022) 139800.

[S10] L. Hang, Y. Sun, D. Men, S. Liu, Q. Zhao, W. Cai, Y. Li, Hierarchical micro/nanostructured C doped Co/Co_3O_4 hollow spheres derived from PS@$Co(OH)_2$ for the oxygen evolution reaction, J. Mater. Chem. A 5 (2017) 11163–11170.

[S11] J. Jiang, J. Xu, W. Wang, L. Zhang, G. Xu, Phosphate ion-functionalized CoS with hexagonal bipyramid structures from a metal–organic framework: Bifunctionality towards supercapacitors and oxygen evolution reaction, Chem-Eur. J. 26 (2020) 14903–14911.

[S12] H. Hu, B. Guan, B. Xia, X.W. Lou, Designed formation of Co_3O_4/$NiCo_2O_4$ double-shelled nanocages with enhanced pseudocapacitive and electrocatalytic properties, J. Am. Chem. Soc. 137 (2015) 5590-5595.
